# Supplementary material for: Observation of phonon Poiseuille flow in isotopically purified graphite ribbons
Source: Nat Commun. 2023 Apr 19;14:2044. doi: 10.1038/s41467-023-37380-5 (PMC10115893; doi:10.1038/s41467-023-37380-5)
Supplement: Supplementary file 1 — Supplementary Information [file 41467_2023_37380_MOESM1_ESM.pdf]

Supporting Information for Observation of phonon Poiseuille flow in isotopically purified graphite ribbons

Xin Huang[1,†], Yangyu Guo[1,†], Yunhui Wu[1], Satoru Masubuchi[1], Kenji Watanabe[2], Takashi Taniguchi[1,3], Zhongwei Zhang[1], Sebastian Volz[1,4], Tomoki Machida[1], and Masahiro Nomura[1,5,*]

[1]Institute of Industrial Science, The University of Tokyo, Tokyo 153-8505, Japan
[2]Research Center for Functional Materials, National Institute for Materials Science, Tsukuba 305-0044, Japan
[3]International Center for Materials Nanoarchitectonics, National Institute for Materials Science, Tsukuba 305-0044, Japan
[4]LIMMS, CNRS-IIS IRL 2820, The University of Tokyo, Tokyo 153-8505, Japan
[5]Research Center for Advanced Science and Technology, The University of Tokyo, Tokyo 153-0041, Japan
[*]corresponding author: Masahiro Nomura (nomura@iis.u-tokyo.ac.jp)
[†]these authors contributed equally to this work

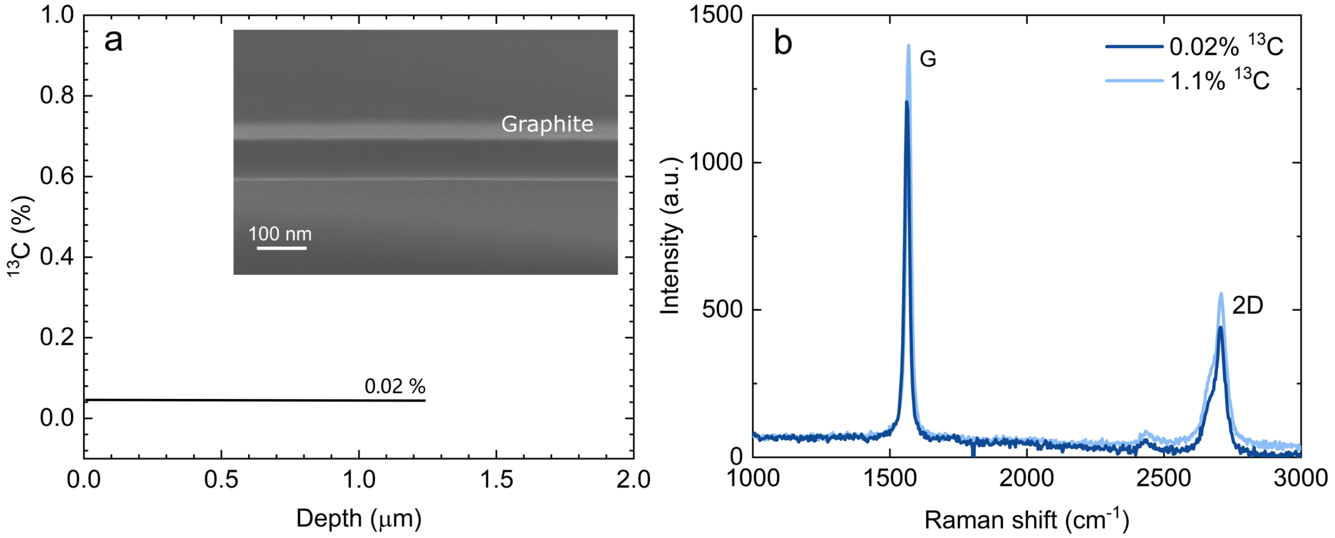

Supplementary Fig. 1. **Sample characterization using TOF-SIMS and Raman spectroscopy. a** ^{13}C concentration measured using time-of-flight secondary ion mass spectrometry (TOF-SIMS). Inset: cross-section view of scanning electron microscope (SEM) image of the suspended isotopically purified graphite ribbon. It indicates the thickness of which is approximately 85 nm. Note that the thickness of natural ribbon is 90 nm, and we assume a negligible thickness-dependence due to the weak van der Waals interaction along the out-of-plane direction as justified in previous works[1,2]. **b** Raman spectra of both isotopically purified (0.02% ^{13}C) and natural (1.1% ^{13}C) graphite samples.

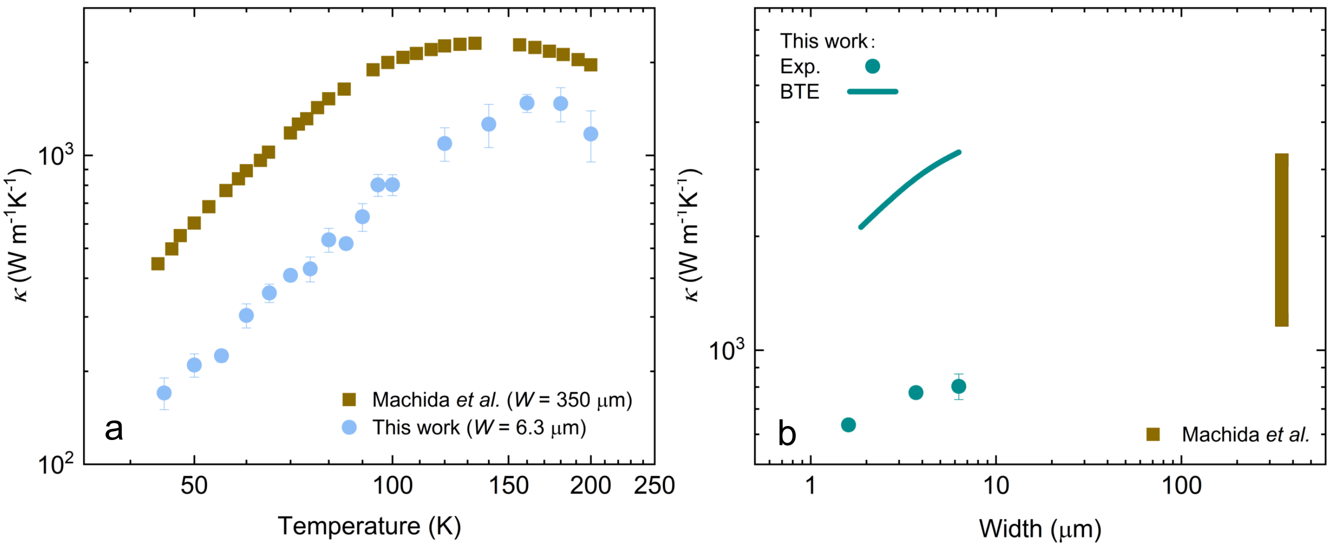

Supplementary Fig. 2. Comparison of in-plane thermal conductivity of submicroscale graphite ribbons with bulk HOPG. a Temperature-dependent in-plane thermal conductivity of our natural graphite ribbon (Length: 30 μm, Width: 6.3 μm, Thickness: 90 nm) and bulk HOPG (Length: 6500 μm, Width: 350 μm, Thickness: 240 μm)[3]. **b** Width-dependent in-plane thermal conductivity of our natural graphite ribbon (Length: 30 μm, Thickness: 90 nm) and bulk HOPG (Length: 6500 μm, Width: 350 μm, Thickness: 8.5−580 μm)[3] at 100 K. Error bars depict the standard deviations of different measurements on the same ribbon. The absolute value of our experimental data is lower than that of our modelling results by BTE with first-principles inputs, which might be caused by the additional resistive scattering of phonons induced by unknown defect or contamination in sample fabrication. However, our experimental width-dependence of thermal conductivity shows good qualitative consistency with our calculated results. Moreover, both experimental and calculated results of $\kappa/G_{\text{ballistic}}$ (as seen in Fig. 4) show very consistent trends to demonstrate the hydrodynamic phonon transport in this work.

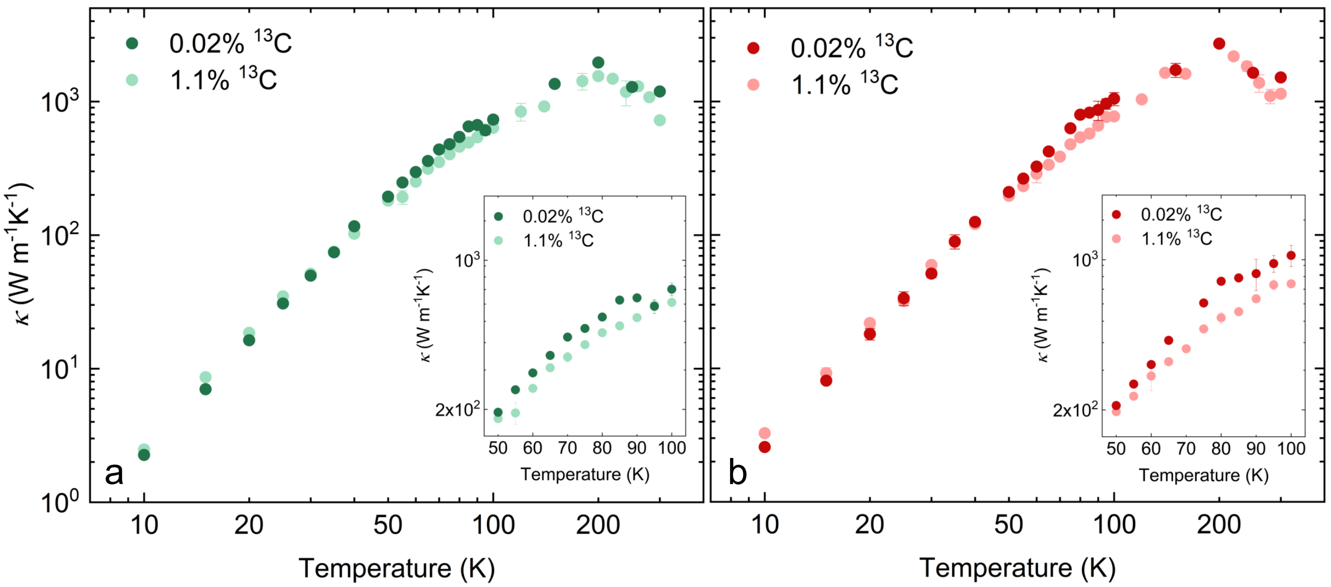

Supplementary Fig. 3. Impact of isotope on the thermal conductivity of graphite ribbons. a,b In-plane thermal conductivity as a function of temperature for both isotopically purified (0.02% ^{13}C) and natural (1.1% ^{13}C) graphite with the designed widths of (**a**) 1.3 µm and (**b**) 3.3 µm. As temperature cools down to 100 K, the weakening of Umklapp scattering causes the dominant isotopic effect, which attributes to the thermal conductivity reduction in natural graphite ribbons (light green and pink dots) compared to that in isotopically purified ribbons (dark green and red dots). Note that the actual widths of these two natural graphite ribbons are 0.3 µm and 0.4 µm wider than that of the isotopically purified ones, respectively, due to the deviation in fabrication, resulting in the minor flip of thermal conductivities at very low temperatures. Insets: thermal conductivity data in linear scale from 50 to 100 K. Error bars depict the standard deviations of different measurements on the same ribbon.

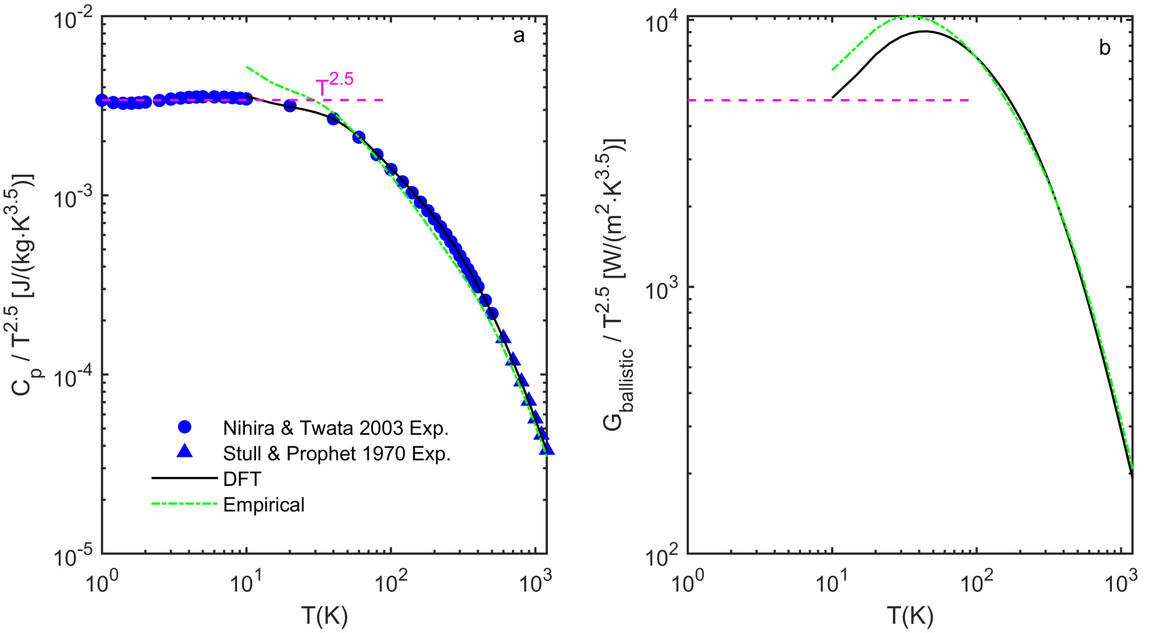

Supplementary Fig. 4. Comparison of thermal properties calculated by DFT (density functional theory) and empirical atomic interaction potential. Temperature scalings of (**a**) heat capacity and (**b**) ballistic thermal conductance of graphite. The discrete symbols represent the experimental data, whereas the solid line and dash-dotted line represents the calculation results by DFT and by empirical atomic interaction potential respectively. The dashed line denotes the low-temperature limit of $C_p \sim T^{2.5}$ and $G_{ballistic} \sim T^{2.5}$. Both theoretical calculations are down to only 10 K due to insufficient resolution of the first Brillouin zone around Γ point below 10 K.

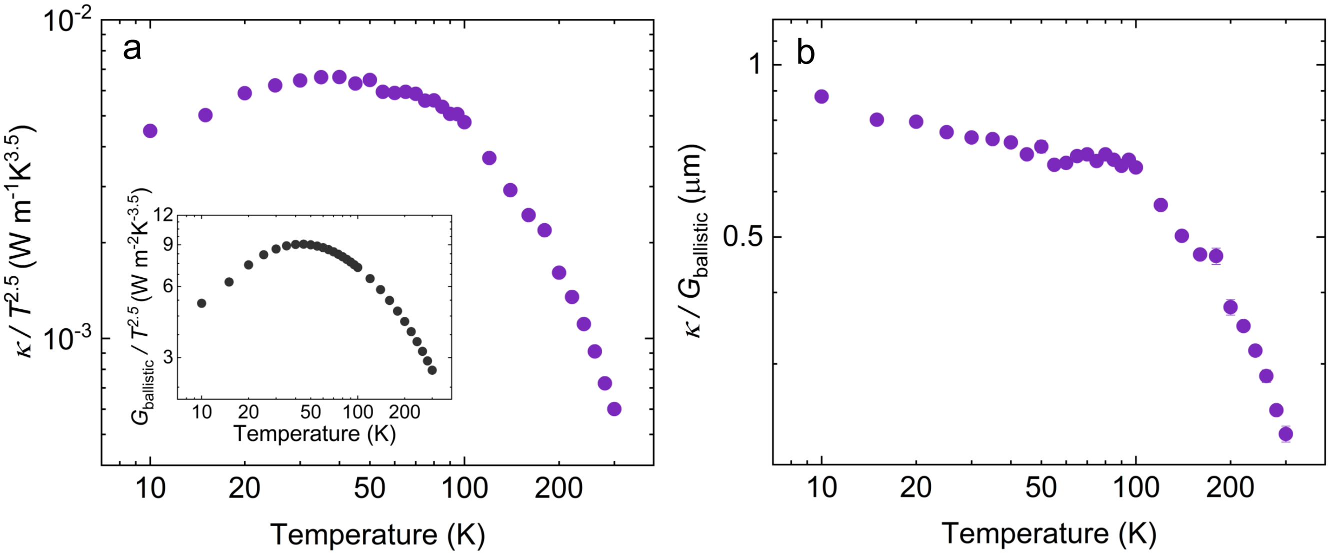

Supplementary Fig. 5. Comparison of the criteria of phonon Poiseuille flow in 500 nm-wide isotopically purified graphite ribbon. a The usual criterion, namely the ratio of thermal conductivity (κ) over $T^{2.5}$ as a function of temperature (T). Inset: temperature-dependence of ballistic thermal conductance ($G_{\text{ballistic}}$) over $T^{2.5}$. **b** The present criterion, namely the ratio of thermal conductivity over $G_{\text{ballistic}}$ as a function of temperature. Error bars depict the standard deviations of different measurements on the same ribbon.

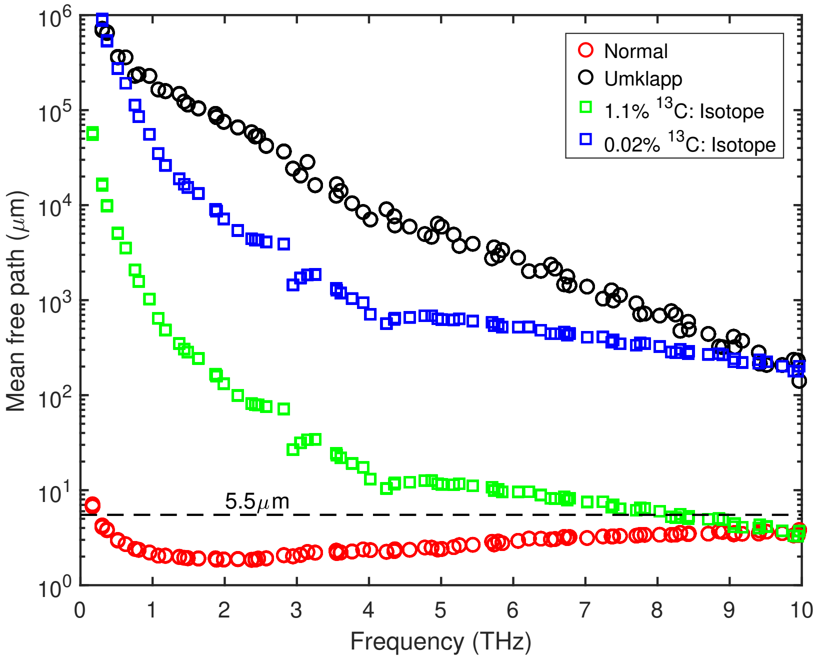

Supplementary Fig. 6. Mean free paths of different phonon scattering processes in graphite at 60 K. The red and black circles denote the mean free path of normal and Umklapp scatterings respectively. The green and blue squares denote the mean free path of isotope scattering in natural (1.1% ^{13}C) and isotopically purified (0.02% ^{13}C) graphite respectively. The results of the bending acoustic (BA) phonons (at $k_z = 0$) which dominate the hydrodynamic transport are shown here. The reference size of 5.5 μm is the sample width of the present graphite ribbon.

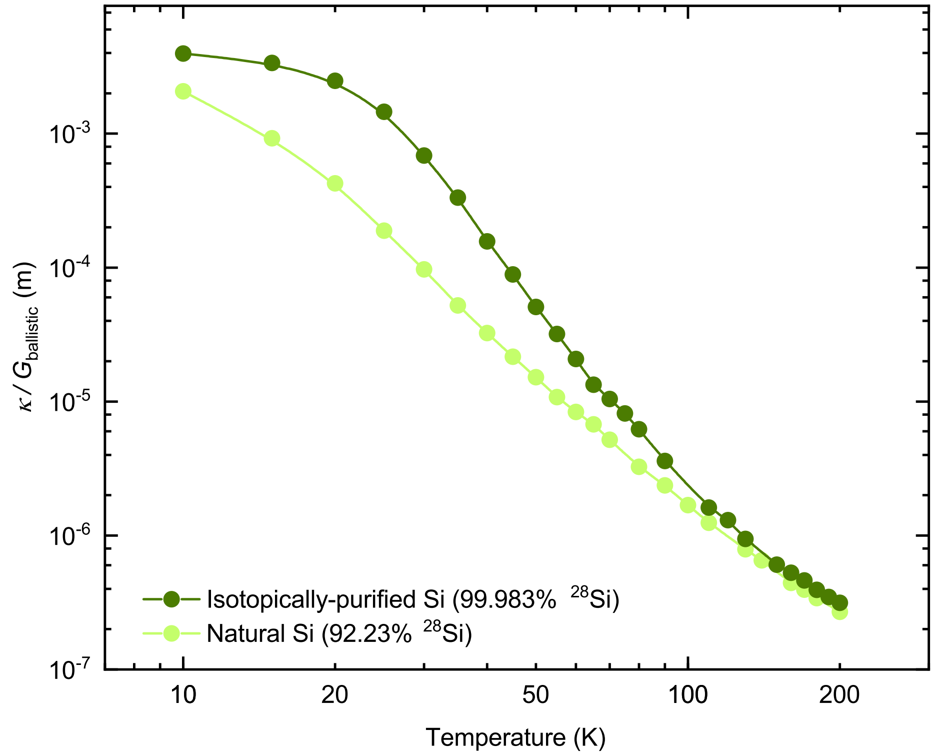

Supplementary Fig. 7. **Examining the criterion of phonon Poiseuille flow in silicon.** The ratio of thermal conductivity (κ) over ballistic thermal conductance ($G_{\mathrm{ballistic}}$) (the present criterion) as a function of temperature for bulk silicon with purified (99.983%) and natural abundance (92.23%) ^{28}Si isotope. The experimental thermal conductivity of silicon is obtained from Ref.[4]. The ballistic thermal conductance for silicon is calculated by the first-principles method.

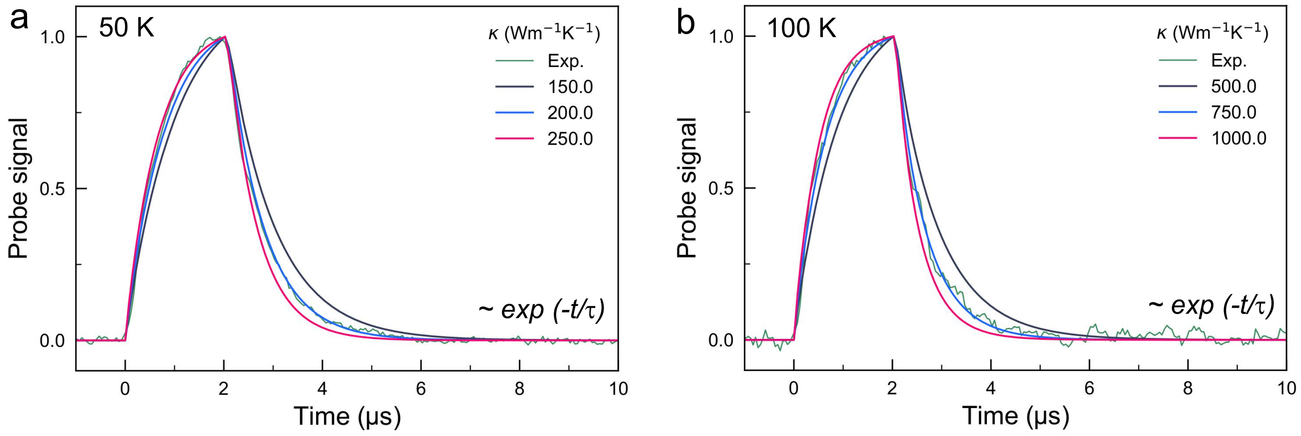

Supplementary Fig. 8. **Fitting experimental data with FEM simulation.** At two typical temperatures of (**a**) 50 K and (**b**) 100 K, we demonstrated the fitting of exponential decay curves obtained in TDTR by the finite element method (FEM) simulations to ensure the accurate extraction of thermal conductivity of our graphite ribbon samples.

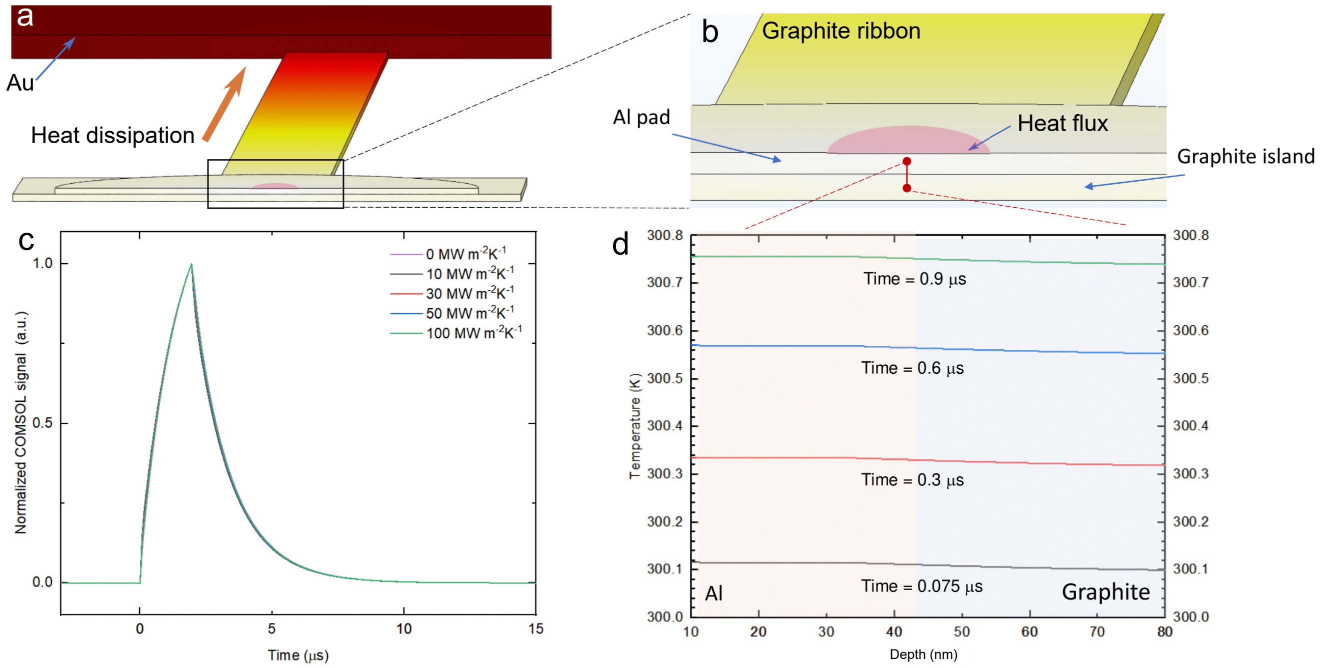

Supplementary Fig. 9. Investigation of thermal boundary conductance between graphite and metals in FEM simulation. a,b Illustration of heat dissipation in FEM model. **c** The dependency of exponential decay on thermal boundary conductance (TBC) between graphite and aluminum. **d** Real-time evolution of temperature distributions around aluminum/graphite interface.

Supplementary Note 1. Sample characterization

Using time-of-flight secondary ion mass spectrometry (TOF-SIMS), we first measure the ^{13}C concentration in our isotopically purified graphite crystals. As shown in Supplementary Fig. 1a, a value of 0.02% is obtained and proves the isotopic purity of the crystals. As our reference sample, we employ natural graphite ("Flaggy flakes") with a naturally occurring ^{13}C abundance of 1.1%, which is the cleanest commercial sample for preparing graphene with large areas.

In addition, we perform Raman spectroscopy to characterize the identical crystal quality of isotopically purified and natural graphite samples. In Supplementary Fig. 1b, the Raman spectra show the same Raman selected G and 2D peaks for both samples. We observe no other initial Raman peaks caused by defects which would destroy the symmetry of carbon hexagonal lattice, and turned out the perfect crystalline for both two graphite samples[5–7]. All the aforementioned sample characterizations confirm that the only difference between the two samples comes from the ^{13}C isotope concentration, and justify the investigation of in-plane thermal conductivity and phonon Poiseuille flow on both isotopically purified and natural graphite samples in this work.

Supplementary Note 2. Examining the criterion of phonon Poiseuille flow in graphite

To have a more quantitative understanding of the satisfaction of hydrodynamic window condition ($l_N \ll W$, $l_R l_N \gg W^2$) in our purified graphite ribbon, we show the MFPs of normal and resistive scatterings of bending acoustic (BA) phonons (at $k_z = 0$) in natural and isotopically purified graphite at 60 K obtained by our first-principles modeling in Supplementary Fig. 6. Note that the resistive scattering here is basically presented by the isotope scattering since the Umklapp process is rare at lower temperatures. In both purified (0.02% ^{13}C) and natural (1.1% ^{13}C) cases, the sample widths are much larger than the MFP of normal scattering ($l_N \ll W$). In the natural graphite ribbon of the present study, the MFP of isotope scattering is around one order of magnitude larger than the sample width (6.3 µm), such that $l_R l_N \sim W^2$. In other words, the graphite ribbon with a natural abundance of ^{13}C does not satisfy the window condition, which is instead valid in the purified ribbon since the MFP of isotope scattering is around two orders of magnitude larger than the sample width ($l_R l_N \gg W^2$). This justifies the present observation of phonon Poiseuille flow in the isotopically purified graphite ribbon with a width of 5.5 µm while not in the natural graphite ribbon with the similar width.

The calculation of the ballistic thermal conductance relies on the used atomic interaction potential. For a comparison, we also calculate the temperature-dependent heat capacity and ballistic thermal conductance of graphite based on an empirical potential[1]. The optimized Tersoff potential and Lennard-Jones potential are adopted for the in-plane and inter-layer interactions respectively. As shown in Supplementary Fig. 4(b), although there is some difference between the absolute values at low temperature, the temperature scaling behaviors of ballistic thermal conductance are almost the same by the first-principles (DFT) calculation and by the empirical potential. Therefore, the theoretical model shall have minor influence on the conclusion of our work. Still we recommend to adopt the first-principles method to calculate the ballistic thermal conductance as the obtained phonon dispersion better reproduces the experimental data of heat capacity, as seen in Supplementary Fig. 4(a).

Supplementary Note 3. Comparison of window conditions for phonon Poiseuille flow and second sound in graphite

A commonly admitted condition of the second sound is that the excitation pulse frequency is smaller than the normal scattering rate but larger than the resistive scattering rate ($\tau_N^{-1} \gg \Omega \gg \tau_R^{-1}$)[8], or is equivalently the dominance of normal scattering over the resistive scattering ($l_N \ll l_{ex} \ll l_R$)[9], with l_{ex} referring to the length of the external excitation. The observation of second sound has been recently reported in the HOPG sample with natural isotope at 100 K[10], followed by a very recent update at 200 K[11] using an improved version of the same technique. In these two studies, the transient thermal grating (TTG) method was used to generate a periodically oscillating temperature field and the decay of the temperature amplitude was measured to indicate the second sound. However, observing the steady-state hydrodynamic phenomenon, namely, the phonon Poiseuille flow, is expected to be more challenging than in the second sound case. As proposed by Guyer *et al.*, phonon Poiseuille flow appears only under the following conditions[9]: $l_N \ll W$, $l_R l_N \gg W^2$. Again, we adopt Supplementary Fig. 6 for a more quantitative understanding of the isotope effect on observing phonon Poiseuille flow and second sound. As demonstrated in the main text and explained in Supplementary Note 2, the phonon Poiseuille flow is only observed in the isotopically purified graphite ribbon with a width of 5.5 µm while not in the natural graphite ribbon with the similar width. For the case of the second sound, the MFP of isotope scattering in the isotopically purified sample and natural

one is around three and two orders of magnitude larger than the MFP of normal scattering respectively, as seen in Supplementary Fig. 6. Hence, the window condition to observe the second sound via TTG with a grating period in-between the MFPs of isotope scattering and normal scattering is satisfied in both purified and natural cases.

Supplementary Note 4. Thermal boundary conductance between graphite and metals

As an unavoidable issue in TDTR measurement, the influence of thermal boundary conductance (TBC) between the aluminum transducer and graphite is also investigated using the finite element method (FEM). We first build a heat dissipation model in COMSOL Multiphysics with the same structures as our μ-TDTR measurement. In the simulation, we apply a Gaussian heat flux pulse to simulate the pump laser focused on the aluminum transducer in the experiment, as shown in Supplementary Figs. 9a,b. Then, by varying the TBC of aluminum/graphite interface, we study its effects on the decay curve and observed a negligible change of decay curves in Supplementary Fig. 9c. To explain, we check the temperature difference between aluminum and graphite layers on the real-time scale after heat flux injection. We find that the aluminum pad and graphite island reached thermal equilibrium within 1μs (Supplementary Fig. 9d) while heat conducts in much longer time-scale through the ribbon. Hence, we conclude a negligible impact of TBC between aluminum and graphite layers on the results in our microsecond-scale experiments.

In the FEM simulation, the 10 μm-long part of the graphite ribbon clamped and attached by the gold heat sink layer is also considered to keep the consistency with the actual experiment. The anisotropic temperature-dependent TBCs (along a-axis and c-axis) between graphite and gold from the literature[12] are adopted in the FEM model to precisely extract in-plane thermal conductivity of graphite ribbon in this work.

References

1. Lindsay, L., Broido, D. & Mingo, N. Flexural phonons and thermal transport in multilayer graphene and graphite. *Phys. Rev. B* **83**, 235428 (2011).
2. Ghosh, S. *et al.* Dimensional crossover of thermal transport in few-layer graphene. *Nat. Mater.* **9**, 555–558 (2010).
3. Machida, Y., Matsumoto, N., Isono, T. & Behnia, K. Phonon hydrodynamics and ultrahigh–room-temperature thermal conductivity in thin graphite. *Science* **367**, 309–312 (2020).
4. Inyushkin, A., Taldenkov, A., Gibin, A., Gusev, A. & Pohl, H.-J. On the isotope effect in thermal conductivity of silicon. *Phys. Status Solidi (C)* **1**, 2995–2998 (2004).
5. Reich, S. & Thomsen, C. Raman spectroscopy of graphite. *Philos. Transactions Royal Soc. London. Ser. A: Math. Phys. Eng. Sci.* **362**, 2271–2288 (2004).
6. Ferrari, A. C. *et al.* Raman spectrum of graphene and graphene layers. *Phys. Rev. Lett.* **97**, 187401 (2006).
7. Eckmann, A. *et al.* Probing the nature of defects in graphene by raman spectroscopy. *Nano Lett.* **12**, 3925–3930 (2012).
8. Lee, S., Broido, D., Esfarjani, K. & Chen, G. Hydrodynamic phonon transport in suspended graphene. *Nat. Commun.* **6**, 1–10 (2015).
9. Guyer, R. & Krumhansl, J. Thermal conductivity, second sound, and phonon hydrodynamic phenomena in nonmetallic crystals. *Phys. Rev.* **148**, 778 (1966).
10. Huberman, S. *et al.* Observation of second sound in graphite at temperatures above 100 K. *Science* **364**, 375–379 (2019).
11. Ding, Z. *et al.* Observation of second sound in graphite over 200 K. *Nat. Commun.* **13**, 1–9 (2022).
12. Duda, J. C., Hopkins, P. E., Beechem, T. E., Smoyer, J. L. & Norris, P. M. Inelastic phonon interactions at solid–graphite interfaces. *Superlattices Microstruct.* **47**, 550–555 (2010).
